# Protein-losing enteropathy as a complication of spontaneous isolated superior mesenteric artery dissection: A case report

**DOI:** 10.1097/MD.0000000000020580

**Published:** 2020-06-19

**Authors:** Xicheng Zhang, Dengqiu Zhao, Zhaolei Chen, Yuan Sun

**Affiliations:** a Department of Vascular Surgery, The Clinical Medical School of Yangzhou University, Yangzhou; b Department of General Surgery, Shanghai University of Medicine and Health Sciences Affiliated Zhoupu Hospital, Shanghai; c Department of Vascular Surgery, The Third People's Hospital of Huizhou, Huizhou, China.

**Keywords:** dissection, protein-losing enteropathy, superior mesenteric artery

## Abstract

**Introduction:**

Protein-losing enteropathy and spontaneous isolated superior mesenteric artery dissection are both rare clinically. Protein-losing enteropathy due to superior mesenteric artery dissection is extremely rare.

**Patient concerns:**

A 46-year-old male with acute abdominal pain and hematochezia was diagnosed with a complete occlusion of the superior mesenteric artery because of dissection. He suffered from diarrhea and hypoproteinemia after an emergency thromboendarterectomy.

**Diagnoses:**

Based on laboratory tests and capsule endoscopy inspection, a diagnosis of protein-losing enteropathy was made.

**Interventions:**

Endovascular treatment was provided.

**Outcomes:**

After stent placement, he quickly recovered without a recurrence of symptoms.

**Conclusion:**

Protein-losing enteropathy is a serious complication of an isolated superior mesenteric artery dissection. Restoring the patency of the superior mesenteric artery is keyed for the treatment of this complication.

## 1 Introduction

Protein-losing enteropathy (PLE) is a rare clinical condition characterized by the excessive loss of plasma protein into the gastrointestinal tract resulting in hypoproteinemia.^[1]^ The etiology can be divided into four categories: erosive gastrointestinal diseases, nonerosive gastrointestinal diseases, mesenteric lymphatic obstruction, and increased central venous pressure.^[2]^ A disorder associated with the superior mesenteric vessel is a rare cause of PLE.^[3,4]^

Spontaneous isolated superior mesenteric artery dissection (SISMAD) is also regarded as a rare condition. To the best of our knowledge, PLE as a complication of superior mesenteric artery dissection (SMAD) has been rarely reported.^[3]^ Here, we present a case who suffered from PLE, resulting from the postoperative SISMAD, and who recovered after endovascular treatment. The patient provided informed consent for this publication.

## 2 Case report

**Figure 1:**
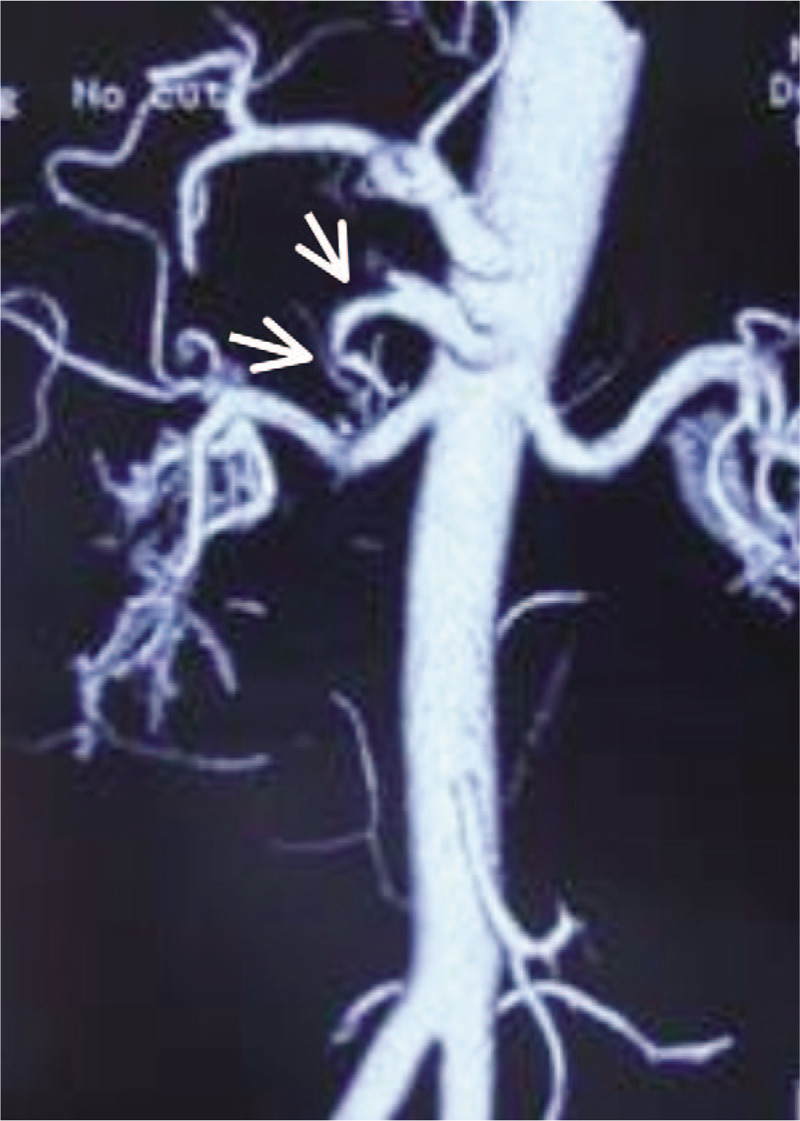
Before the operation, computed tomography angiography (CTA) revealed that superior mesenteric artery (SMA) had occluded completely (white arrow).

A 46-year-old man, who presented with a 4-h history of acute abdominal pain, nausea, vomiting, and hematochezia, was admitted to our emergency department. He had no history of trauma, abdominal pain, etc and his blood pressure is normal (125/76 mm Hg). Contrast-enhanced computed tomography angiography (CTA) showed that the superior mesenteric artery (SMA) was completely occluded due to dissection (Fig. 1). Laboratory tests revealed a normal white blood cell count of 8.9 × 10^9^/L (4.0–10.0 × 10^9^/L) and normal albumin level: 42 g/L (35–50 g/L). Two hours after admission, he underwent an emergency exploratory laparotomy because of persistent abdominal pain and hematochezia. During the operation, the entire small intestine was pale and no palpable pulses in the SMA trunk could be detected. The true lumen was completely compressed by the thrombotic false lumen. A proximal thrombectomy and endarterectomy were performed, and the distal intimal flap was fixed to the wall of the SMA. The pulsation of the SMA and the color of the small intestine were restored after this process.

**Figure 2:**
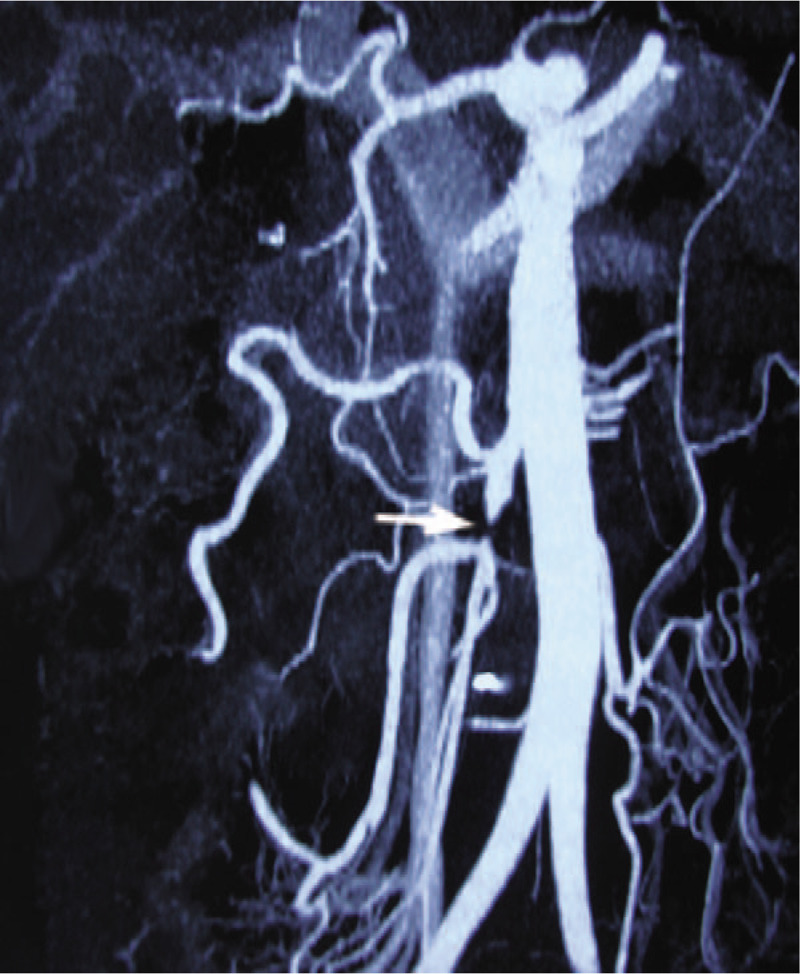
One month after the operation, computed tomography angiography (CTA) showed the main superior mesenteric artery (SMA) trunk remained occluded for about 1 cm (white arrow), but the collateral pathway can supply the small intestinal branches of the superior mesenteric artery.

**Figure 3:**
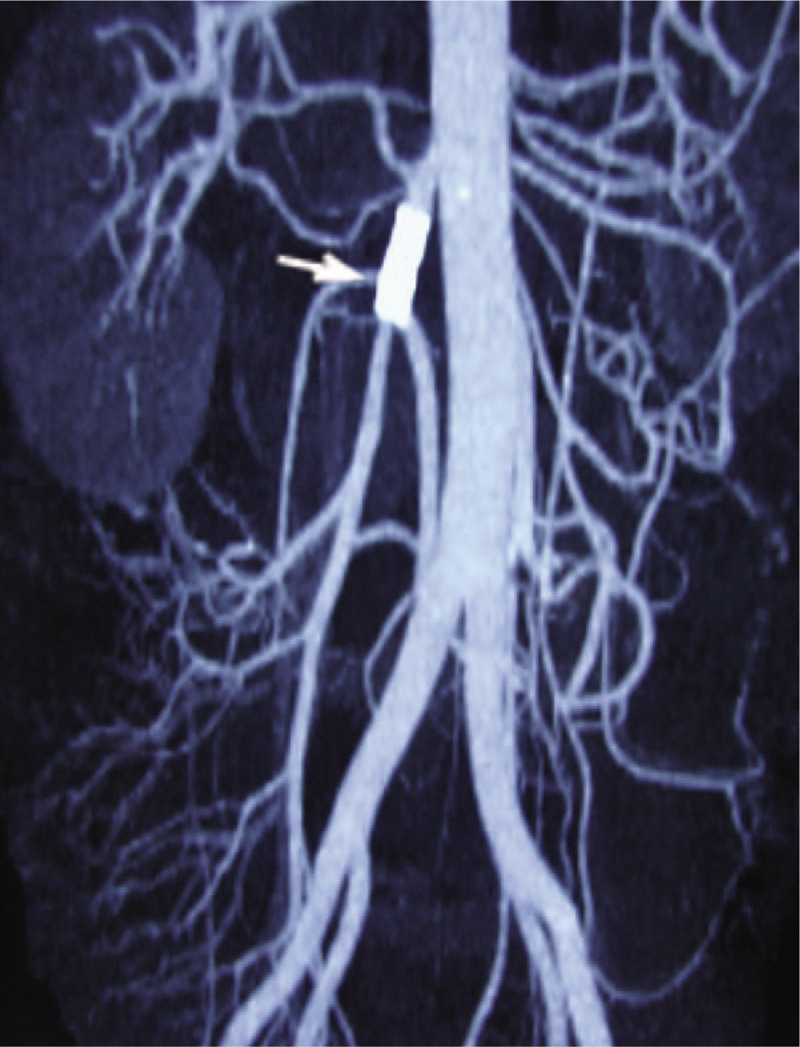
Six months later, follow-up computed tomography angiography (CTA) examination revealed stent patency without stenosis (white arrow).

The patient's abdominal pain disappeared soon after the surgery, but he suffered from severe diarrhea (about 9–10 times a day). Although the diarrhea symptoms were controlled to 3 to 5 times a day by conservative treatment, his serum albumin level began to continuously drop. He was given a vasodilator, albumin, and nutritional support through intravenous infusion every day to avoid severe hypoproteinemia. After 30 days, his albumin level fell to around 13 to 18 g/L. The CTA showed that the branches of the SMA were patent but the SMA trunk was occluded by about 1 cm (Fig. 2). Capsule endoscopy revealed small intestinal mucosal erosions and showed a clear distinction between diseased intestinal mucosa and normal mucosa. Quantitative determination of albumin in urine was normal, and no other abnormal loss of protein was detected. A balloon-expandable stent (6 × 18 mm, Cordis, USA) was implanted in the occlusive segment. The patient recovered uneventfully with no recurrence of diarrhea or abdominal pain and his albumin level rose to around 34 g/L one month later. A follow-up CTA showed that the stent was patent and in the correct position 6 months later (Fig. 3).

## 3 Discussion

PLE is a rare cause of hypoproteinemia in the clinic. In healthy individuals, protein loss through the intestinal epithelium plays a tiny role in total protein loss. If no other causes of hypoproteinemia can be identified, then diagnosis of PLE should be considered. The clinical manifestations of PLE are variable, which are related to the underlying cause. However, hypoproteinemia contributes to the primary symptoms, such as edema and malnutrition.^[2–4]^

Only a few cases of SMAD have been reported previously, but the cases in China have significantly increased in recent years.^[5]^ Choosing the best treatment for SMAD is not definitive thus far.^[6]^ Most patients can be managed by conservative treatment. However, emergency surgery or endovascular treatment is necessary for the patients suffering from impending arterial rupture or bowel gangrene.^[6]^

For this patient, the occlusion of SMA after the operation was perhaps because the swollen flap was lifted by blood flow to form a valve that obstructed the blood flow. Fortunately, the collateral branch avoided the necrosis of the intestine. However, the blood supply of the collateral circulation was apparently not sufficient and the bowel was in a chronic ischemia condition, which led to the erosion of the mucosal epithelium. The intracellular permeability increased and tight junctions between cells became wider. The erosions of the intestine mucous membranes had been shown by the capsule endoscopy. Inevitably, the continuously protein-rich fluid loss from the eroded epithelium to the gastrointestinal tract would lead to diarrhea and hypoproteinemia.

PLE caused by SISMAD is rare. Refractory hypoproteinemia is a clinical feature of this disease. This case had a history of SMAD dissection, and CT showed segmental SMAD occlusion, indicating the possibility of ischemic lesions in the intestine. Capsule enteroscopy confirmed the segmental ischemic erosion of the small intestinal mucosa. The above features support the diagnosis of PLE. In addition, a small intestinal mucosa biopsy or (99 m) Tc-labeled albumin imaging can help in the diagnosis, but the latter is not often used clinically.^[7]^

Treatment options for PLE should be directed at the underlying condition. However, if edema, ascites, malnutrition, or micronutrient deficiency is present, intravenous albumen supplement and nutritional treatment are necessary.^[2]^ Regarding the reported cases of PLE caused by mucosal erosion, many of them underwent enterectomy of the pathological segment.^[2–4]^ The cause in this case was intestine ischemic due to SMAD, and the first vascular reconstruction had not completely corrected the intestinal ischemia. Therefore, endovascular therapy is preferred to restore blood flow.^[3,6]^ The balloon-expandable stent was chosen because it was easy to locate and does not cover more branches. After the stent placement, the erosion of the mucous could be repaired slowly and the serum albumin increased continuously. Therefore, for these types of postoperative patients with chronic intestinal ischemia, endovascular treatment is a simple, safe, and effective optional measure.

## Author contributions

**Conceptualization:** Xicheng Zhang.

**Data curation:** Yuan Sun.

**Investigation:** Yuan Sun.

**Supervision:** Dengqiu Zhao.

**Validation:** Zhaolei Chen.

**Writing – original draft:** Xicheng Zhang, Dengqiu Zhao.

**Writing – review & editing:** Xicheng Zhang, Yuan Sun.
